# Exploring prioritization through systematic literature surveys and case studies

**DOI:** 10.1186/s40064-015-1320-0

**Published:** 2015-09-22

**Authors:** Varun Gupta, Durg Singh Chauhan, Kamlesh Dutta

**Affiliations:** Department of Computer Science and Engineering, National Institute of Technology, Hamirpur, India; GLA University, Mathura, India

**Keywords:** Reprioritization, Prioritization, Mass market software development

## Abstract

The software development process is a complex process, especially when the software has to be released in a phased manner. The high stakes involved and several constraints on resources lead to the selective implementation of the user requirements at each stage of the development. If the requirements considered, do not fit well, they have to be reprioritized. The objectives of this paper are to create a background related to the area of reprioritization and to create a distinguishable position from the prioritization area. The paper elaborates on the current state of reprioritization practices adopted in the software industry. The gaps in research in the area of reprioritization to present future opportunities for the research community are also analysed. This paper builds on the findings of a systematic literature survey (to analyze state of the art in the area of reprioritization), previous case studies (to gain understanding of real scenarios through limited available information), and more detailed real case study (focussed on reprioritization alone) conducted by fewer multinational software development organizations. Based on our studies it is concluded that the efficient reprioritization methods are required to be adopted in software engineering practices of the organisation in order to sustain in the highly competitive mass market.

## Background

Mass market development progresses from a limited set of requirements, gathered from “unsure and small” customer base through increments, facing flood of requirements as input from “larger and slightly known customer base”, until the product captures market and becomes stable. Progress in calendar time leads to the discovery of new viewpoints of new customers [including continuous feedback from existing customers (Karlsson et al. 2002)] and new insight into the development process and market trends (Carmel and Becker 1995; Dahlstedt et al. 2003; Potts 1995; Lubars et al. 1993), thereby making the change in requirement priorities as a function of time; a usual activity. The particular increments will involve the stakeholder requests for the new functionality and the requests for the changes to be made in already delivered functionality. The decision is to be made regarding the requirements to be selected for the next release, which makes it common for new changed and delayed requirements to fight for their place. Already implemented requirements are to be resubjected to prioritization because priority varies, as a function of time and it is better to keep priorities updated for many good reasons (example priorities employed during regression testing).

Organizations employ prioritization techniques based on pairwise comparisons to establish priorities, which involve huge effort, as number of input requirements increases because every requirement is to be compared with each other; again leading to increase in number of pairwise comparison (Berander 2007). Prioritization of new requirement will lead to comparison with already implemented, changed and delayed requirements leading to re-estimation of the priorities of the latter. A re-allotment involves replacement of old priority with newly calculated priority, meaning complete prioritization again, which is natural with pairwise comparison. The less effortful method is required that will quickly and accurately estimate and re-estimate priorities of requirements.

Reprioritization impacts various activities of incrementally developed software like decision aspect prioritization, requirement prioritization and regression testing (Gupta et al. 2012a, b, 2014a). Reprioritization technique finds its applications in a variety of activities, such as reprioritization of services in IEEE 802.11p (WAVE) VANET’s (Salahuddin et al. 2013), reprioritization of messages in real-time vehicle applications (Preston 2012), reprioritization of presentation materials (Allen et al. 2010), reprioritization of test cases (Budnik and Subramanyan 2013), reprioritization of projects (Heldman 2006) etc.

The area of reprioritization demands the work to identify unaddressed issues and problems. The broad scope of reprioritization study is selected to focus on setting the right position with respect to prioritization, motivating audiences towards the need for generating optimal reprioritization practices and adopting practices in development processes.

The paper is structured as follows: “Research questions” sets the context of the paper by setting research questions with an aim to analyze the state and practices of reprioritization in mass market situations. “Methodology” elaborates the findings of the systematic literature survey and compares it with the general practices adopted by the mass market software developing firms (details in “State of art: an outcome of systematic literature survey and case studies”). “Reprioritization in context to prioritization” tries to set a different, yet related context of reprioritization with respect to prioritization. Details of a literature survey and case study findings and comparison are carried out in “State of art: an outcome of systematic literature survey and case studies”. Finally, the paper concludes in “Conclusion and future work” highlighting the need for considering reprioritization-aware development so that efficient and evaluated reprioritization practices can benefit the overall development.

## Motivation

The survey, conducted by Yoo and Harman (2012) highlights the limitations of existing prioritization techniques (especially those based on ratio scale) to handle the flood of requirements as they suffer from scalability problem (with increase in number of requirements, effort increases). Number of requirements will keep on increasing which will increase prioritization efforts. Techniques based on other measurement scale can be used to drastically lower efforts, but the less powerful scale limits this idea because it does not provide detailed information about the results for further analysis. The most powerful ratio scale techniques are based on pairwise comparison (example AHP). These are considered to be more accurate than the non pairwise ones (Karlsson 1996; Karlsson et al. 1998; Perini et al. 2009); although it suffers greatly from scalability problems (Achimugu et al. 2014; Voola and Babu 2013; Perini et al. 2009; Ahl 2005; Karlsson et al. 1998, 2004; Lehtola and Kauppinen 2006; Ribeiro et al. 2011). Software Engineers face dilemma of tradeoffs between the measurement scale, working principle (pairwise and non pair wise) and effort to be invested.

The literature lacks the prioritization methods that can handle large number of requirements with minimal efforts. Literature provides many prioritization techniques differing in measurement scale used and working algorithm, etc. but the efficiency in mass market development environments (continuous flood) is yet to be completely established. The available work is limited to models for performing reprioritization activities, and only a few reprioritization methods, actually focusing on the flood of requirements, are available. Such reprioritization methods must be employed on real softwares to properly comment on their likelihood of passing uncertainty and risks involved in mass market developments.

The case studies with software development organizations (Gupta et al. 2014b) reveal the mercy situation of software industries, where few industry people understand the importance of changing priorities, yet they do not have any established means of doing so, while the majority of them are still unmotivated towards reprioritization. The industries are still using prioritization techniques for changing priorities of changed requirements, while the new requirements are allotted priorities using same prioritization techniques based on guesswork and Business Values.

This motivated the authors of this paper to come up with a comparative analysis based on systematic survey of literature and software industry case studies, that are focused on lack of reprioritization based research and industry software development practices. The net outcome may make the audience aware of the need for sound reprioritization especially based on pairwise comparisons.

## Research questions

The presented work aims to explore areas of reprioritization to analyze how much this area is seen differentiable from the area of prioritization in literature and practice. This exploratory research in the area of reprioritization aims to uncover various issues and problems that prove costly to software industries involved in highly competitive mass market developments. The literature survey aims to find answers to the following research questions:

1. How is the area of reprioritization treated with respect to the area of prioritization?
2. How is reprioritization carried out by mass market software industries?
3. How do the current reprioritization activities handle flood of requirements in mass market development situations?
4. How is the dynamism in the priorities of decision aspects handled in mass market developments?

## Methodology

With the objective of finding the answers to above mentioned research questions, the paper adopts the following research methodology:

- Performing systematic literature surveys to gain insights into the reprioritization area with the objective of uncovering the practices and issues with respect to mass market developments.
- Gaining insights into the current reprioritization practices of software developing organizations by conducting interviews with their representatives.
- Comparison of the literature analysis with those extracted from the current practices analysis.

## Reprioritization in context to prioritization

The objectives of this paper are as follows: first, to create background related to the area of reprioritization for creating the distinguishable position from the prioritization area (throughout the paper); second, to present the current state of reprioritization practice in software industries (through interviews with multinational companies), and third, to analyse the research gaps in the area of reprioritization so as to present future opportunities for research community (comparison between literature survey and case studies).

The research framework provides the comparison of the two processes i.e. prioritization and reprioritization.

The authors of this paper define reprioritization as “The process of re-establishing the relative ordering among the individual elements of a continuously updated set of requirements by involving the updated decision aspects and updated list of stakeholders”.

This area re-executes prioritization algorithm on a requirement set of larger cardinality (as compared to the cardinality of previous increment) within the environment of enhanced uncertainties and larger cardinalities of inputs involving requirements and decision aspects of a large number of identified customers. This area is best illustrated mathematically as below:

For increment “I”, let R be set of requirements to be implemented. |R| represents cardinality of the set which further represents the number of requirements to be implemented.

Let S be the set of stakeholders to be involved in decision makings and D be the set of decision aspects. |S| and |D| represents cardinalities of representative sets.

For increment “J” with J > I i.e. I precedes J in implementation ordering, the new sets are denoted by R1, S1 and D1.

Clearly, |R1| ≫ |R|, |S1| ≫ |S|, |D1| ≫ |D|.

Let ORDER() be the methods that creates the implementation ordering among the requirement set.

Heuristic() is the method that guides the prioritization process in optimal manner. Optimization may involve reduction in efforts, time and the increase in quality of decision making.

The reprioritization is achieved using reprioritization() methods. Control abstraction of the reprioritization() function is given below:



These are the control abstractions meaning that actual implementations are not specified. There are various ways of implementing the ORDER() and Heuristic() function. Heuristic() function reuses the old priority to estimate new priority. New priority can be used to estimation of priority of new requirement. Working of this function is based on working algorithm of selected technique.

Further, the cardinality of set R1 keeps on increasing thereby increasing the prioritization efforts and finally increasing reprioritization complexities.

**Fig. 1:**
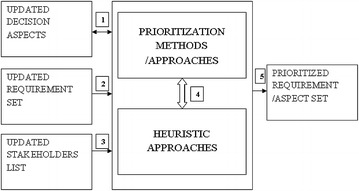
Reprioritization framework

The above reprioritization process is given in the form of a framework in Fig. 1.

Figure 1 shows the reprioritization framework. The prioritization methods or approaches are used to create ordering among requirements. The incremental mass market software development experiences flood of requirements; hence re-execution of prioritization methods is infeasible solution. These methods are to be supplemented with few heuristics or changes in order to manage the scalability problems that may occur with increase in number of requirements.

Decision aspects are also prioritized and their priorities may change. The list of decision aspects may alter thus demanding complete reprioritization (block numbered 2). The list of requirements is updated due to the discovery of new customers that specifies new requirements and changes. The new set of requirements comprises new, changed, delayed and already implemented requirements; all of them subject to prioritization. Prioritization techniques find it difficult to handle such large number of requirements. To uncover this problem the heuristic approach is used to lower prioritization effort. An example of heuristic approach as proposed in (Gupta et al. 2014a) employs the numerical assignment technique to quickly prioritize the available requirements (denote with set R). The pairwise comparison is performed among fewer requirements of set R and all newly emerged and changed requirements. Lesser effort is due to less effort for reprioritization although new and changed requirements are completely pairwise prioritized. Further, minimization is possible if the new and changed requirements are prioritized with minimal efforts.

Double arrow numbered 1 indicates that aspects are employed for reprioritization and are also subjected to reprioritization. The result is a reprioritized list of requirements or decision aspects.

## State of art: an outcome of systematic literature survey and case studies

The third objective of this paper is to analyse the research gaps in the area of reprioritization. The gaps will convince the researchers about the possibilities in the area of reprioritization by analyzing the work available in current literature (especially case studies) and the findings of real case studies with multinational software development organizations. This section of the paper does this job by sub diving into subsections that includes the following:

- Systematic literature surveys will help to gain understanding of the re-prioritization area by analyzing the already done research work. To perform the systematic literature survey, few popular bibliographic databases were triggered against search string, which yielded six papers related to research questions framed in “Research questions”. These fetched papers had been successful in coming up with conceptual models describing the manner reprioritization takes place, factors affecting decision making during reprioritization and gaps existing between current practices and those described in the literature. Few papers like (Gupta et al. 2013, 2014a, b) are not covered in the systematic literature survey, although they were published well before the date of searching the bibliographic databases and the journals are well covered in ACM digital library and Scopus. The reason might be that the papers were not indexed at the time of search in above mentioned bibliographic databases.
- The reprioritization practices of software development firms were analyzed by performing interviews with their software engineers, who participated as their representatives. The outcome is finally compared with the literature survey findings.

These are discussed in great detail as below.

### Systematic literature survey

The systematic literature survey was conducted to better position the readers of this paper in the area of reprioritization. Systematic survey resulted in extraction of less number of informative papers from various bibliographic databases due to limited work done in the area. These papers had been able to come up with conceptual models that focus on how reprioritization is done and the factors that help in decision makings. These conceptual models are derived from analysis of literature and refined as a result of interactions with the software developing firms representatives. The finally derived model represents the industrial practices and highlights the gaps with current work in the area of reprioritization/prioritization. The systematic literature survey is conducted by querying bibliographic databases and finally extracting out meaningful papers from the list.

The guidelines for performing systematic literature survey as disseminated in Kitchenham and Charters (2007) are followed to perform literature survey. The below mentioned subsections describe the process.

#### Research method

The review of literature analyzes current trends in the process of requirement reprioritization and focuses on open research issues in this area.

To analyze the literature, the following digital libraries/databases are searched against the following query string i.e. “reprioritization of requirements” on 15th August, 2014.

- IEEE Xplore.
- Springer Link.
- Science Direct.
- ACM Digital Library.
- Taylor and Francisco.
- Wiley Online Library.

The triggering of the above databases with this bigger string is because some researchers use prioritization and reprioritizations terms interchangeably.

The trigger of the databases against above mentioned string yielded total 618 research papers with few repetitions in the set. The majority of the papers does not cover the area of reprioritizations, and thus are not capable of answering any of the formulated research questions.

The final number of papers was only 09, after analysis of research paper titles, keywords, abstracts and full texts.

The number of papers extracted from each bibliographic database is given in Table 1. Analysis was carried out considering the Table 2. This set was reduced to 06 after the removal of repetitive papers in the bibliographic databases (Table 1).

**Table 1:** Papers selected after process “a”

Bibliographic databases	Papers selected after process “a”	Repeated papers
1. Science Direct	01	01 (available in ACM also)
2. IEEE Xplore	02	01 (available in ACM also)
3. ACM Digital Library	05	03 (one each with IEEE, Springer and Science Direct)
4. Springer Link	01	01 (available in ACM also)
5. Wiley	00	None found answering any of the four research questions
6. Taylor & Francisco	00	None found answering any of the four research questions

**Table 2:** Inclusion and exclusion criteria

Inclusion criteria	Exclusion criteria
1. The papers must be answering at least one research question 2. Papers related to reprioritization alone 3. Papers (both short and long) related to the case study, review (if any), methods, tools and techniques of the reprioritization	1. Papers unable to answer any research question 2. Papers related to prioritization 3. Papers related to some other area like prioritization with small proportions (like paragraphs) related to reprioritization, exception being the above papers that discuss new methods, techniques etc. related to reprioritization

**Fig. 2:**
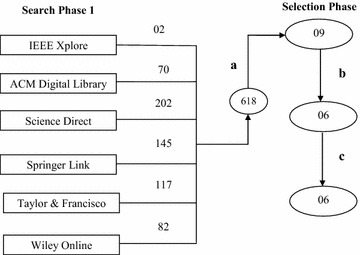
Search process for literature survey

The final set of obtained papers is subjected to analysis. The series of pruning of research papers is illustrated with the help of Fig. 2.

In this figure, the label “a” denotes the transformation applied on the collected papers after analysis of abstracts, keywords and the text. Label “b” removes duplicates, “c” analysis the references as reported in the papers left after process labelled “b” i.e. duplicates finally removed.

#### Details of extracted papers

The systematic literature survey yielded six papers. Three more related papers, which were not identified at the time of survey due to non-indexing in bibliographic databases at the time of literature search, are also included for necessary analysis. Among these three papers, two papers were related to reprioritization techniques, third one is related to case study results of prioritization and reprioritization practices. This paper will consider all these papers as extracted papers in upcoming sections. The details of extracted papers are as given below:

The authors (Racheva et al. 2008) identified the main players of the prioritization process and various problems that confront it. In order to meet the above goals, the literature is searched for various requirements of the prioritization models that are employed in agile software developments. After the analysis of the collected information, the factors that are considered by the clients and those that affect various types of decision making like Business Values, project constraints, learning during project, effort estimation etc. during requirement reprioritizations are identified and presented in the form of conceptual models.

Finally the various prioritization issues are identified and presented with the suitable solutions. Some of the issues addressed are:

- The authors stressed on the fact that how will the Business Value for each requirement be computed. Possible suggested solutions include weight allotments, consideration of dependencies, modelling of criteria, need of current implementation.
- Another issue is that it might be impossible to compute Business Value for few requirements. Possible solutions include: (1) relating such requirements to those that create maximum Business Values (2) considering the time dependency criteria between such requirements.
- Third issue relates to quantity related requirements. For such requirements, the computation of the Business Values would be quite difficult since they cannot be separated for other requirements. By considering quality requirements as functional ones, one could be able to target this problem.
- The fourth issue relates to dynamism in Business Values. Such values change for each increment, so a method should be there to compute the new value of the requirement by considering variations in Business Values.
- Another issue related to the decision is the selection of an appropriate prioritization techniques. In other words, how a software engineer chooses suitable requirement prioritization techniques among the list of many techniques? Solutions include the consideration of various criteria, like number of requirements, stakeholders, volatility levels etc.
- The decision regarding setting of the scope of next increment is also one of the issues related to reprioritizations.

In their paper, Racheva et al. (2010a), tried to find answer of the research question, “What are the key concepts to consider when prioritizing the requirements from clients perspectives in agile projects”? The various factors affecting and guiding the clients during performing reprioritizations related decisions are identified by analysis of the analysis of literature. Various agile journals and digital libraries like IEEEXplore, ACM, Google Scholar, Interscience, Citeseer, Agile Journal, Dr. Dobb’s and InfoQ methods are searched and the citations are also used as the source. The results were analyzed through the abstracts and conclusions leading to the selection of 42 papers. It was found after analysis of these papers that total 22 of them highlight requirement of prioritization techniques. The application of Grounder Theory (GT) yielded various findings that were illustrated graphically with the help of two conceptual models denoted by A and B.

Model A gives the course grained description of the reprioritization of requirements in agile developments for successive increments. For each increment, the requirements are reprioritized and then few of them with higher priority are selected for the current implementation in the sprint. Sprint backlog does not encounter update during particular sprint while it might be possible that developers are able to implement few requirements of the sprint backlog. These pending requirements are then added back to prioritized product backlog. The new requirements might arrive and update the above backlog, this backlog is then reprioritized and the same process is repeated until all increments are delivered.

Model B gives the fine grained description of the decision making that happens during reprioritization. It highlights the various factors that are considered by the clients during taking decisions regarding reprioritization. The authors reported that Business Values, risks, effort or size, learning experience and external changes are five aspects employed during reprioritization.

Business Values are estimated for all requirements by the clients. Negative values or penalties, importance or relative importance are also considered as Business Values. Risks or uncertainties are also considered during reprioritization decision making. Risks, costs and requirement size are computed by developers, but considered by clients that work in consultation with the developers.

Project constraints include release dates, budget, velocity etc. Only those requirements are implemented that satisfy the current resources held by developing firms. Learning is the main activity in agile software developments which happens at both individual and on team levels. External environment encounters change due to which the requirement priorities also change. Few of the examples includes competitor’s activities, change in user needs, business rules etc.

Racheva et al. (2010b) presented the conceptual model of reprioritization. It highlights the course grained description of decision making that happens at inter iteration time. The questionnaires followed by interviews with total 11 agile software development participants, i.e. project managers, developers, product owners, clients and scrum masters. The case studies of 10 projects were considered during the interviews of these experts.

Bakalova et al. (2011) tried to answer the following research question, “Which concepts of agile prioritization are shared in practice and in literature, and how are they used to provide guidance for prioritization”. The authors tried to analyze the gaps between the current trends in reprioritization as described in the literature and those described in real life software developments in agile components. The conceptual model suggests that there are seven aspects which are considered by the clients while making reprioritization decisions. The conceptual models are slightly modified after the further insights brought by the participants and dependencies were finally added as the seventh aspect. To analyze the gaps, the concepts created as a result of multiple agile case studies, are searched in the description of requirement prioritization methods identified through literature surveys. If the concepts are missing from requirement prioritization techniques, it means that literature does not adapt well to the current industrial settings. To analyze the above information, the results fetched after a search in the requirement prioritization descriptions is organized in the form of a table. Analysis of this table is reported by the authors as follows:

- Existing requirement prioritization techniques provide the descriptions at every course grained level, i.e. descriptions are not in detailed fashion.
- Existing techniques do not employ all the concepts as identified by the authors through multiple case studies.
- Existing techniques do not give a detailed description of how to perform prioritization, or the information about factors affecting decision aspects, and stakeholders to be involved etc.

Daneva et al. (2013) carried out a survey to uncover different aspects related to the process of prioritization at inter iteration time, i.e. reprioritization. The survey was conducted in the form of interviews with the people working on live large agile projects, which the authors had termed as Alpha, Beta and Gamma. The main findings of the paper (only those related to answering of the research questions as formulated in the paper) are as given below:

- The organizations employ Business Values, Requirement Dependencies, Volatility, Risk, Effort, and Technical Debt as the criteria for reprioritization.
- Maturity of the organization, the way domain knowledge is shared, and the use of delivery stories are recognised as factors responsible for affecting the manner the reprioritization is carried out.

The paper has not discussed any method for performing reprioritization but has explored concepts related to the reprioritization practices in large scale projects. The discussions with project practitioners discussing the typical roles on client and vendor side and prioritization criteria gives little picture of reprioritization process as adopted in the firms. This may partially answer our second research question, yet our aim was to explore the method in great detail. Further, the paper also proves that the firms do not prioritize/reprioritize the decision aspects as employed during reprioritization. This answers our research question number 4.

Kukreja and Boehm (2013) proposed a two step prioritization model that is capable of performing prioritization and reprioritization. It involves prioritization of business goals followed by decomposition of software into Minimal Marketable Features (MMFs) which are further prioritized with respect to prioritized goals. Each MMF is decomposed into requirements; requirements are stated as Win conditions and are further prioritized. Reprioritization involves just changing priorities of MMFs as priorities of Win conditions will automatically change as per change in priority of parent MMF. The process model is not evaluated on mass market live software and thus makes it hard to determine that how much improvement it will yield if employed for reprioritization with flood of requirements. So, this paper will not be considered as a model provided for mass market developments. But it provides answers to first two research questions of this paper.

Gupta et al. (2014b) had given the results of the survey that was conducted with few multinational software development organizations. The survey results revealed that the organizations agreed to the fact that it is not the decision aspects, but the requirements which area associated with ever changing priorities, i.e. decision aspects were not considered as candidates for reprioritization because decision aspect prioritization was considered an unheard-of phenomenon. Organizations wanted a cost effective solution for performing reprioritization to stay in markets offering less costly products.

Gupta et al. (2013) proposed a multilayered dynamic approach for performing reprioritization. The technique handles a different category of requirements differently. For example, the paper uses “Average Density” to reprioritize delayed requirements. New and changed requirements are handled in a different manner. This technique is demonstrated with the help of live system of the Library Management Software and is suitable for both agile and non agile developments. But the techniques based on pairwise comparisons are exceptions and this technique may not be able to handle reprioritization of pairwise compared requirements beyond some threshold value.

Gupta et al. (2014a) handled the problem of the inability of reprioritization methods to reprioritize pairwise compared requirements. The technique employs the numerical assignment technique to quickly prioritize the available requirements (denoted with set R) and performing pairwise comparison among fewer requirements of set R and all newly emerged and changed requirements. The technique is evaluated on a real case study of the “Tool for automatic analysis and comparison of different release planning methods”. Results proved that the technique had good coverage on the entire set of requirements and establishes priorities using minimum number of pairwise comparisons.

The papers as disseminated by authors in Racheva et al. (2008, 2010a, b), Bakalova et al. (2011), Daneva et al. (2013) give conceptual models and do not provide any method to perform reprioritization, while those disseminated in Kukreja and Boehm (2013), Gupta et al. (2013, 2014a), present new reprioritization techniques.

The papers related to conceptual models are analysed in Table 3 and papers related to new reprioritization techniques are analysed in Table 4.

**Table 3:** Literature work of papers related to conceptual models

S. no.	Paper reference	Findings and deliverable
1.	Racheva et al. (2008)	The paper identified various issues in prioritization; factors governing and influencing the client’s decision making during reprioritization are presented. Few solution strategies are also given by the authors
2.	Racheva et al. (2010a)	Conceptual model of reprioritization is built on the basis of survey of literature. Factors influencing the reprioritization decision making are identified and is considered as a refined form of those identified earlier
3.	Racheva et al. (2010b)	The conceptual model as created above is further refined as a result of interactions with the industry participants. This is because of the insights brought by the software development firms
4.	Bakalova et al. (2011)	The conceptual models as created above are further refined as a result of more series of interviews with software development firms. “Dependencies” was the new factor to be added. These conceptual models are considered true reflections of industrial practices. The seven identified factors are searched in the prioritization description in literature to identify gaps. These gaps provide future directions for research work
5.	Daneva et al. (2013)	Carried out a survey to uncover different aspects related to the process of prioritization at inter iteration time, i.e. reprioritization. This paper identified the different roles as played by different people at client and vendor side, reprioritization criteria’s adopted, factors affecting manner reprioritization is carried out

**Table 4:** Literature work of papers presenting new reprioritization technique and case studies

S. no.	Paper reference	Findings and deliverable	Future work/limitations
1.	Gupta et al. (2014b)	Decision aspects reprioritization is not undertaken by organizations, but they do recognize the need for handling requirements. Staying in markets by reducing the development efforts (especially time and cost) is one of the criteria that make them reluctant for reprioritizing already prioritized requirements. This aims to get insight into prioritization and reprioritization of both decision aspects and requirements. Updated documentation is another finding of the case study	The area of reprioritization is explored to finer levels by interactions with organizations that had been using agile and non agile models from the time since back. The interactions should only focus around reprioritization Although the presented case study had been done with mass market software development organizations and had flavoured the tastes of both agile as well as non agile software developments
2.	Gupta et al. (2013)	Technique for performing reprioritization of new, changed and delayed requirements	Not evaluated on real case studies May not be suitable for reprioritization of pairwise comparison based requirements beyond some value of a number of requirements
3.	Gupta et al. (2014a)	Technique to perform reprioritization of pairwise compared requirements with minimal efforts It has good coverage of requirement set and involves minimal pairwise comparisons	Should be extended to reprioritize decision aspects Should be executed on evolutionary software with thousands of requirements and continuous flood of requirements
4.	Kukreja and Boehm (2013)	This paper provides a two step process model for performing prioritization and reprioritization. Reprioritization is facilitated by automatic re-computation of requirement priorities by changing the priorities of parent MMF as per changing business environment	Techniques not evaluated on real mass market situations like ever increasing requirements, aspects and stakeholders

### Synthesis of literature findings

The papers extracted from literature are discussed in previous section and summarized in Tables 3 and 4. The extraction must be synthesized so as to generate answers to the four research questions (“Research questions”).

1. How is the area of reprioritization treated with respect to the area of prioritization?
  All the extracted papers are related to reprioritization activity, with few giving conceptual models and the remaining presenting new techniques. Conceptual models and new techniques establish the difference between reprioritization and prioritization. The papers given in Table 3 do not highlight the problems that confront the reprioritization activities. Possible unidentified problems could be as follows:
  The conceptual models are silent on considering already prioritized requirements for reprioritization. Gupta et al. (2012b) reported that there is a direct impact between requirement prioritization and regression testing. To achieve the objective of lesser costs attributable to minimization of regression testing, the requirement priorities should reflect the current market trends.
  The conceptual models are silent on reprioritization involving a change in priorities of decision aspects. This is due to the finding of Gupta et al. (2014a) that decision aspects are subjected to reprioritization to have accuracy in implementation set of the requirements.
  Reprioritization should focus on the views of developers as many non functional requirements are better prioritized by the developers rather than the clients. Svensson et al. (2011), after the analysis of eleven software developing firms, reported that prioritization of quality requirements are a neglected activity. Quality requirements are taken as low priority requirements and the main stress is on implementing functional ones.
  The reprioritization techniques must focus on minimizing the reprioritization efforts. An increase in the efforts mean increase in delivery time and costs. An increase in the values of these parameters is not acceptable in either bespoke and market driven developments. The reprioritization techniques have to execute on the larger set of inputs, than those operated in earlier increment. The reprioritization process should be able to handle large number of requirements with minimal efforts, i.e. free from scalability problem.
  There are no guidelines/results that could help in decision making for selection of particular prioritization technique for performing reprioritization.

The remaining papers (given in Table 4) present reprioritization techniques that need to be evaluated on real software so that their response against large requirement sets and large number of clients could be evaluated.

RQ 2. How is reprioritization carried out by mass market software industries?
  Table 3 shows the generic process for performing reprioritization without reference to the mass market development. The reference to mass market situations, large stakeholders, requirements etc. are missing for the models. Table 4 gives generic techniques for performing reprioritization which may or may not be applicable for mass market developments after numbers of increments. In Gupta et al. (2014a) and Kukreja and Boehm (2013) are the two techniques evaluated on real software but not at higher values of requirements. These techniques can be considered as mass market techniques with the requirement of stronger evaluations at higher values of inputs. The techniques as given by Gupta et al. (2014a) and Kukreja and Boehm (2013) were based on performing reprioritization with an aim of minimization of parameter like efforts.

RQ 3. How do the current reprioritization activities handle flood of requirements in mass market development situations?
  Gupta et al. (2014a) and Kukreja and Boehm (2013) could be considered as mass market techniques but need strong evaluations. The authors of both the papers have not discussed that how their techniques are well acceptable against flood of requirements.

RQ 4. How is the dynamism in the priorities of decision aspects handled in mass market developments?
  None of the technique relates itself with reprioritization of decision aspects. The techniques are specially mentioned for requirements and their applicability for reprioritization of decision aspects is still an unaddressed issue. Hence the literature survey is unable to reveal any work related to reprioritization of decision aspects.

The Overall literature lacks reprioritization methods. The work that could be identified in the literature does not get mapped to the practical realities of mass market developments like large requirements, stakeholders and aspects. The above synthesized observations are presented in Table 5. Table 5 excludes the case study work given in Gupta et al. (2014b) because it does not provide any new reprioritization technique.

**Table 5:** Synthesis of literature survey finding

S. no.	Paper references	New reprioritization proposal				Establishes reprioritization vs prioritization
		Requirements	Decision aspects	Based on mass market	Based on parameter minimization	
1.	Gupta et al. (2013)	No	No	No	No	Yes
2.	Gupta et al. (2014a)	Yes	No	Yes	Yes	Yes
3.	Racheva et al. (2008)	No	No	No	No	Yes
4.	Racheva et al. (2010a)	No	No	No	No	Yes
5.	Racheva et al. (2010b)	No	No	No	No	Yes
6.	Bakalova et al. (2011)	No	No	No	No	Yes
7.	Daneva et al. (2013)	No	No	No	No	Yes
8.	Kukreja and Boehm (2013)	Yes	No	Yes	Yes	Yes

### Reprioritization survey results

The objective of performing the case study is to deeply consider the practices of the software developing organizations from the perspective of reprioritization alone. The interviews were conducted with the software engineers of Eight Multinational software development companies. The interviews were conducted face to face through personnel meetings.

The researchers respect the privacy terms of the industries agreed upon at the time of interview and the information is shared in such a manner that it does not disclose their identities.

All the eight companies are reputed software organizations with development centres and customer bases almost all over the world. Most of these organizations have many branches not only in different parts of world but India as well.

Most of the companies have higher average annual return and 10,000+ employs in single Indian site. Many of the employees are, as reported by the interviewed professionals, software engineers.

A number of software engineers, who were interviewed, had worked on sufficient number of projects at various levels. Abstract details per organization are as given in Table 6.

**Table 6:** Multinational organization details

S. no.	Organization	Reprioritization techniques		
		Software engineers employed	Interviewed engineers	Engineers’ characteristics
1.	A	10,000+	07	Experience of working for the development of many projects at current and some other organizations performing roles and responsibilities. Higher level engineers were also involved
2.	B	10,000+	04	Experience of working for the development of many projects at current and some other organizations performing roles and responsibilities. Higher level engineers were also involved
3.	C	10,000+	03	Experience of working for the development of many projects at current and some other organizations performing roles and responsibilities. Higher level engineers were also involved
4.	D	6000+	02	Experience of working for the development of many projects at current and some other organizations performing roles and responsibilities. Higher level engineers were also involved
5.	E	400–600	02	Experience of working for the development of many projects at current and some other organizations performing roles and responsibilities. Higher level engineers were also involved
6.	F	400–600	01	Experience of working on many consultancy based projects; typically those which require small team sizes
7.	G	200–400	02	Intermediate experience with software development projects
8.	H	500–1000	01	Experience of working on many consultancy based projects; typically those which require small team sizes

The interviews were aimed to gain insights into mass market incremental software development process by focussing on prioritization and reprioritization practices. During the interviews, the authors focused on prioritization practices, because the industry uses some other terminology that stands for something else in literature. To be in a better position to answer RQ 1, it was felt better to let industry representatives answer questions for both prioritization and reprioritization.

1. How is the area of reprioritization treated with respect to the area of prioritization?
  The interview sessions revealed that organizations are using prioritization practices. It means that the reprioritization is seen similar to prioritization. The organizations are performing prioritizations, i.e. allotting values of priorities to new and changed requirements only so that the whole process may reduce costs, time and effort. This will be treated as parameter based prioritization and not reprioritization.

RQ 2. How is reprioritization carried out by mass market software industries?
  As both prioritization and reprioritization are seen similar thus the only way to know how it is performed is just by looking at the manner the software industries re-prioritizes backlog and requirements prioritized earlier.
  The organizations do not reprioritizes previously prioritized requirements and the main stress is on changed and new requirements. The chances of a backlog item getting into implementation is rare, if it happens then it is only the compliance with Business Values and some guess work that does the job for software engineer. The organizations are carrying out prioritizations and not reprioritizations.

RQ 3. How does the current reprioritization activities handle flood of requirements in mass market development situations?
  Organizations do not have method to prioritize and even reprioritize flood of requirements. When talked about flood of requirements, most of them replied that “Short sprints make them possible to handle large number of requirements”. It is again the compliance with Business Values and some guess work that makes software engineer to select highest priority requirements.

RQ 4. How is the dynamism in the priorities of decision aspects handled in mass market developments?
  Decision aspects are fixed and neither are the priorities allotted nor changed. Decision aspects does not update it with the passage of time. They are not reprioritized. The organizations employ Business Values, stresses on reusability and consider advice of developers, i.e. the guess work of developers, which is consistent with the finding of Gupta et al. (2014b). Decision aspects are neither prioritised not reprioritized. So the question of the updation of decision aspect does not arise.

The Interview findings are summarised in Table 7.

**Table 7:** Interview sessions findings

S. no.	Organization	Reprioritization techniques			
		Requirements flood	Decision aspects	Based on mass market	Based on parameter minimization
1	A	No	No	Not exactly	Satisfaction of Business Values
2	B	No	No	Not exactly	Satisfaction of Business Values
3	C	No	No	Not exactly	Satisfaction of Business Values
4	D	No	No	Not exactly	Satisfaction of Business Values
5	E	No	No	Not exactly	Satisfaction of Business Values
6	F	No	No	Not exactly	Satisfaction of Business Values
7	G	No	No	Not exactly	Satisfaction of Business Values
8	H	No	No	Not exactly	Satisfaction of Business Values

The interactions with the industry personnel gave additional insights as mentioned below:

- The industries have a fixed small customer base. The advice of such customer base (representation of market trends) is taken during development of each increment of software. Mass market developments suffer from the problem of unknown customer base. The organization does not have any stakeholder identification technique.
- The organizations do not have any limited value of the number of requirements that could be best served by the prioritization techniques. During interactions, the authors aimed to know that even as organizations employ prioritizations, do they feel some value of the number of requirements as “unmanageable”? Such a number may give some threshold value, up to which the prioritization finds its place for reprioritization. Averaging such values obtained from different companies help to even know better the threshold. Unfortunately such a relation could not been derived.

### Comparative results of literature survey and case studies

The findings of the interview sessions (Table 7) reveal the same situation as revealed by literature findings (Table 5) except for RQ 1, the software industry is in dire need since any mistake or use of some weak practices may wipe out a company from the competitive market. In market customer satisfaction matters can be mapped directly to their requirements; indirectly to unstated requirements like quality requirements and definitely delivery time and cost. The comparative analysis is mapped to different research questions below and summarized in Table 8.

**Table 8:** Comparison of literature work and interview session findings

S. no.	Parameter of interest (availability)	Interview sessions findings	Literature survey findings	Whether finding consistent with from literature findings
1	Identification of stakeholders	No	No	Yes
2	Sound reprioritization techniques	No	No	Yes
3	Decision aspect reprioritization	No	No	Yes
4	Parameter minimization based reprioritization techniques	No^a^	Yes^c^	Yes
5	Mass market based reprioritization	No^b^	Yes^c^	Yes
6	Reprioritization of already prioritized, changed, backlog and new requirements	No	Yes^c^	Yes
7	Applicability of prioritization technique for reprioritization	No	No	No

^a^Reprioritization is simply prioritization in case of organizations. So it will be better if it is called parameter minimization based prioritization

^b^As discussed earlier, consideration of the mass market is only partial

^c^Limited techniques are available. This represents the need for further research in the area

1. How is the area of reprioritization treated with respect to the area of prioritization?
  The literature has limited papers available on reprioritization. Comparison of Figs. 1 and 2 shows the limited work is going in the area of reprioritization. Although to a limited degree, the available papers establish prioritization VS reprioritization situation, i.e. at least positions reprioritization in separate position than prioritization. The interview sessions revealed that organizations employ prioritization techniques for ranking new and changed requirements only throughout the life cycle of the software. Limiting the set of requirements (being limited to new and changed only) make ranking somewhat manageable but the priorities of other requirements will remain outdated. The organizations are afraid of employing requirement priorities during phases like regression testing. The guess work will result in investment of large costs since costs need to be invested in providing better customer feedback support, incorporation of feedbacks and no optimization in priority based activities like regression testing.

RQ 2. How is reprioritization carried out by mass-market software industries?
  The organizations consider only new and changed requirements during development of each increment. Such requirements are ranked by using Business Values, reusability, minimization of cost and time and some other guess work by developers. Literature provides, with limited reprioritization, techniques that needs evaluation on large number of requirements against changing aspects. These techniques provide good solution up to number of requirements and the performance drastically degrades thereafter. Neither the literature nor the industries have sound technique for identification of stakeholders that are identified mostly in later increments during incremental mass market developments. Both the literature and industry lack the sound and trustworthy reprioritization techniques that could work well in mass markets. Industrial practices are prioritization based while techniques available in literature have still to prove the ability to work with large numbers.

RQ 3. How the current reprioritization activities handle flood of requirements in mass market development situations?
  Industrial practices are prioritization based while techniques available in literature have still to prove the ability to work with large numbers. In case of industry, prioritization is based on guesswork and fixed aspects. Mistakes done could be corrected in next sprint as per almost all industry recipients. In case of literature, the available techniques have to prove the ability on flood of requirements.

RQ 4. How is the dynamism in the priorities of decision aspects handled in mass market developments?
  Industry prioritization practices are based on fixed aspects while reprioritization practices do not provide any discussion of whether they could be applied on reprioritization of aspects as well. Decision aspects are neither prioritized nor reprioritized in industry while literature techniques need evaluation when aspects of reprioritization are to be validated.

## Conclusion and future work

**Fig. 3:**
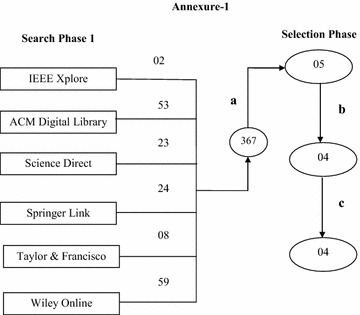
Search process for literature survey for the year 2012. Comparison of the Figs. 2 and 3 shows that even after 2 years, the number of papers related to this area of reprioritization has not grown too much (just 3 papers).The papers produced are negligible in number as compared to the papers published in other areas. This shows the negligence and lack of research in this important area

The area of reprioritization is new and unexplored area that lacks research addressing the ways of performing this activity with minimal efforts. We consider the reason for the limited research the lack of papers that better positions this activity apart from prioritization. The same evidence came from the comparison of the literature survey findings for 2 years, i.e. 2012 and 2014 (Figs. 1, 2), done using the same string, i.e. “reprioritization of requirements” against same bibliographic databases. Comparison of the Figs. 2 and 3 shows that even after 2 years, the number of papers related to the area of reprioritization has not grown very much (just 3 papers). The papers produced are negligible in number when compared to the papers published in other areas.

This paper aims to provide the research community a convincing arguments in order to make research reprioritization oriented. The limited research in this area is collected through systematic literature surveys and analyzed to gain better understanding of the area.

The paper discusses the results of the interactions done with software development firms that aim to get deeper into the area of reprioritization alone. The findings of the interactions are compared to those of analyzed through literature findings. The comparison shows similarities which provide convincing arguments for conducting more research in this area. The problems which have been identified are similar to those which have been collected through by Gupta et al. (2013) but the figures and understandings as presented in this paper are detailed and highlight things minutely, because the survey and interactions are only focussed on reprioritization.

The overall analysis of this paper gives new directions to future research that addresses the following:

- Efficient reprioritization methods capable of handling large number of aspects and requirements with minimal efforts are the need of the hour. Efficiency means that reprioritization should also minimize the parameters like cost, time and efforts etc. A large number of aspects and requirements mean that the technique should be capable of working in mass-market development scenarios.
- Effective identification of stakeholders is important thing to be done in mass-market development, as the actual customer base is unknown in beginning increments. Better, the customer base better is the development. Effective identification is not there in the practices of the organization.
- The applicability of existing prioritization methods during reprioritization is an unaddressed issue.
- Evaluation of effective reprioritization methods on real mass market case study in the near future, encouraging the organizations to invest more efforts to minimize burden on reprioritizations and minimizing prioritization efforts during reprioritizations.
- Overall impact analysis of reprioritization techniques on time, cost and quality of software incremental and overall development is interesting to watch.

The practices of organizations are not effective enough to handle the dynamism in mass market developments especially failing in proper selection of stakeholders, handling changing priorities associated with decision aspects and large number of requirements. They cannot depend on available literature as literature also provides limited and partially validated support in the area of reprioritization.

Much more evidence came from the comparison of the literature survey findings for 2 years, i.e. 2012 and 2014, done using the same string, i.e. reprioritization of requirements against the same bibliographic databases.
